# Impact of 3T multiparametric MRI and FDG-PET-CT in the evaluation of occult primary cancer with cervical node metastasis

**DOI:** 10.1186/s40644-016-0097-x

**Published:** 2016-11-04

**Authors:** Mária Gődény, Zsolt Lengyel, Gábor Polony, Zoltán Takácsi Nagy, Gergely Léránt, Orsolya Zámbó, Éva Remenár, László Tamás, Miklós Kásler

**Affiliations:** 1 Department of Diagnostic Radiology, National Institute of Oncology, Ráth György street 7-9, Budapest, 1122 Hungary; 2 Pozitron Diagnostics LTD, Hunyadi János street 9, Budapest, 1117 Hungary; 3 Department of Otorhinolaryngology, Head and Neck Surgery, Semmelweis University, Szigony u.36, Budapest, 1083 Hungary; 4 Department of Radiotherapy, National Institute of Oncology, Ráth György street 7-9, Budapest, 1122 Hungary; 5 Head and Neck Surgery Department, National Institute of Oncology, Ráth György street 7-9, Budapest, 1122 Hungary; 6 Department of Postgraduate Education and Scientific Research, University of Medicine and Pharmacy, Tirgu Mures, Romania

**Keywords:** Magnetic resonance imaging, Positron emission tomography, High-field MRI, Diffusion-weighted imaging, Carcinoma of unknown primary origin

## Abstract

**Background:**

This study aimed to determine the ability of multimodal evaluation with multiparametric 3T-MRI (MPMRI) and positron emission tomography - computed tomography (PET/CT) to detect cancer of unknown primary origin (CUP) with neck lymph node (LN) metastasis.

**Methods:**

The study group comprised 38 retrospectively analysed consecutive patients with LN metastasis in the head and neck (HN) region without known primary tumours (PTs). Statistical values of 3T-MRI and of FDG-PET/CT scans were evaluated.

**Results:**

Of the 38 CUPs, conventional native T1-, T2-weighted and STIR sequences detected 6 PTs. Native sequences plus diffusion-weighted imaging (DWI) found 14-, and with fat suppression contrast-enhanced T1-weighted measurement as well as with the complex MPMRI found 15 primaries and with PET/CT 17 CUPs could be evaluated, respectively. The detection rates were 15.8, 36.8, 39.5, 39.5 and 44.7 % for conventional native MRI, native plus DWI, native with contrast-enhanced MRI (CE-MRI), for MPMRI, and for PET/CT, respectively. The overall detection rate proved by histology was 47.4 %. PET/CT provided the highest sensitivity (Sv: 94.4 %) but a lower specificity (Sp: 65.0 %), using MPMRI (Sv: 88.2 %) the specificity increased to 71.4 %. DWIincreased specificity of the native sequences (Sp: 76.2 %). Conventional native sequences plus DWI as well as 3T-MPMRI and PET/CT were same accurate (Acc: 79.0 %) and had similar likelihood ratio (LR: 3.42, 3.03 and 2.62) in detecting unknown PT sites.

**Conclusions:**

The accuracy of FDG-PET/CT and MPMRI in case of CUP in finding the primary cancer in the neck regions is identical. While using PET/CT whole body information can be obtained in one examination. MPMRI shows the local soft tissue status more accurately. In cases of CUP PET/CT should be the first method of choice if it is available. MPMRI can clarify the exact primary tumor stage, and it can be advantageous in clarifying the prognostic factors, which is necessary in case of advanced tumor stage and when surgery is under consideration. In case low N stage is likely after the clinical examination and *wait and see policy* can be considered, MPMRI is recommended, and in this case the significance the of radiation free MPMRI is increasing.

**Electronic supplementary material:**

The online version of this article (doi:10.1186/s40644-016-0097-x) contains supplementary material, which is available to authorized users.

## Background

According to the European Society for Medical Oncology (ESMO) guidelines, cancers of unknown primary origin (CUP) represent a heterogeneous group of metastatic tumours for which a standardised diagnostic work-up fails to identify the site of origin at the time of diagnosis. The term CUP is reserved for epithelial malignancies because other types of malignancies (such as melanomas, sarcomas, lymphomas) can usually be easily identified using immunohistochemistry [1, 2]. CUPs account for 3–5 % of all malignancies [3, 4] and in 24–36 % of patients with CUPs, the metastatic lymph node (LN) manifestations of the unknown primary cancer are in the head and neck (HN) region [5]. The primary tumour (PT) site in these patients is eventually identified in only 20–40 % of cases [6].

In general, the prognosis of CUP is poor, and the median survival time for such patients is 6–10 months. In cases of CUP in the HN, the outcome is more favourable, with 5-year survival ranging from 35 to 50 %; this is especially true in cases with detected PTs because targeted therapy is possible (PT surgery, radiation focused to the tumour) [7].

The majority of cancers in the HN are squamous cell carcinomas (SCC), followed by adenocarcinoma, undifferentiated carcinoma, and adenoid cystic carcinoma.

The location corresponding to the cytological result of metastatic LNs may provide an indication of the site of the PT. Finding, localising, and staging the PT and the cervical nodal metastasis play key roles in treatment planning [8].

For the accurate diagnosis of CUP, physical examination, fibre-optic laryngoscopy, and nasopharyngoscopy are applied, although the majority of suspected primary sites are detected by radiographic workup.

PET combined with CT (PET/CT) scanning improves the detection of occult cervical lymphatic disease and assists in the localisation of unknown PT and distant metastases [9]. A meta-analysis of 16 studies (published in 2004) found that FDG-PET or PET/CT detected 74 PTs (24.5 %) in 302 CUP patients with neck node metastases; the overall sensitivity was 88.3 %, specificity 74.9 %, and accuracy 78.8 % [10]. Moreover, FDG-PET/CT has lower specificity than sensitivity and is more useful for detecting distant metastases and synchronous tumour than LN metastasis [11, 12].

Functional and molecular information can also be obtained using magnetic resonance imaging (MRI), as diffusion-weighted MRI (DW-MRI) is able to characterise the tissue based on differences in tissue water mobility. In a recently published paper, it was concluded that analysis using diffusion-weighted imaging (DWI) could be very useful to differentiate malignant and benign lesions in the HN region [13, 14]. The ADC is a quantitative biomarker of the extent of diffusion restriction, measured in 10^−3^ mm^2^/s units, although no common threshold ADC value exists in clinical routine to differentiate between malignant and benign tissue [15–17]. The first significant paper showing the usefulness of DWI in HN tumours was published in 2001. Wang et al. used an ADC value threshold of 1.22×10^−3^ mm^2^/s to differentiate benign from malignant tissue, and this value demonstrated 86 % accuracy, 84 % sensitivity and 91 % specificity for predicting malignancy [18].

By performing a retrospective analysis, we sought to evaluate new imaging methods in the management of occult tumours. In particular, we compared the ability of positron emission tomography - computed tomography (PET/CT) and 3-Tesla (3T) multiparametric magnetic resonance imaging (MPMRI), using high-resolution anatomic and functional DWI, to detect tumours in our CUP patient population with cervical node metastasis.

## Methods

We retrospectively reviewed the medical records of all patients with a diagnosis of neck LN metastasis of CUP, who underwent 3T MPMRI with conventional as well as DWI and whole-body fluoro-2-deoxy-D-glucose (FDG)-PET/CT examinations between July 01, 2012 and May 15, 2016. Thirty eight patients (24 male, 14 female, mean age 63 years) fulfilled the following inclusion criteria: 1) the diagnosis of metastatic LN in the neck was confirmed with fine needle aspiration biopsy (FNAB) and 2) 3T MPMRI and FDG-PET/CT imaging were performed within 6 weeks prior to treatment except for two patients who had 7 and 8 weeks before treatment. All patients were first evaluated with complete head and neck physical examinations performed by HN specialists. All patients had carcinoma proven by cytology after FNAB from a cervical LN metastasis before PET/CT and MRI.

For this study, 2 radiologists and nuclear medicine physicians specialised in HN imaging evaluated the MPMRI and PET/CT images retrospectively. Clinical findings were used to evaluate the performance of both methods. The two experienced interpreters evaluating MPMRI data sets were in consensus, FDG-PET/CT interpreters were also in consensus. One of the MPMRI interpreters has 20 years of experience in the head and neck tumor cases, the other one has 10 years of experience. One of the FDG-PET/CT interpreters has 10 years of experience in the field of head and neck cases, the other one has 6 years of experience. The evaluating interpreters were blinded to the other imaging modality. Postgraduate courses are frequently held in head and neck radiology in our institute, with constant consulting with the PET/CT center. The observers reviewed all visible LNs and the possible PT sites in all 38 patients. We considered tumour-positive imaging results as true positives when the second clinical examination with panendoscopy and biopsy revealed the PT.

MPMRI was performed using a 3T wide-bore MR scanner (General Electric Discovery 750w, Milwaukee, WI) using a high-resolution HN surface coil. We optimised our MR techniques to increase spatial resolution and maintain an adequate signal to noise ratio (SNR), and the scan time was minimised to avoid motion artefacts. T1W and STIR axial and T1W coronal sequences were applied to the whole neck extended the latest till tracheal bifurcation using also axial T1W slices to the upper mediastinum. We applied a field of view (FOV) of 240 × 240 mm in axial and 280–320 mm in coronal, slice thickness of 5 mm, 0.5–1 mm interslice gap, and 288 × 256, 320 × 288 matrix size with nex2. T2W turbo spin echo and DW echo-planar axial images were obtained to the regions of suspected PT with FOV 240 × 240, slice thickness/gap of 3.6/0.3 mm. The DW sequence was performed as part of our routine examination before the contrast-enhanced T1-weighted with fat suppression (CET1WFS). A 2D spin-echo DW echo-planar STIR imaging sequence (TR 6130/TE minimum, 65–70 ms, inversion time 200 ms) was used an acquisition matrix of 112 × 80 or 64 × 84, numbers of acquisition of 3, 6, 12 depending on the “b” values. Quantification was performed using an apparent diffusion coefficient (ADC). To reduce the influence of the perfusion effect on the ADC calculation, we applied higher b-values (50, 500, and 1,000 s/mm^2^). Regions of interest (ROIs) were placed in the typical soft tissue signal intensity parts of the measurable lesion, which showed high signal intensity on the *b* = 50 and *b* = 1,000 images and low signal intensity on the ADC map.

After the administration of an intravenous gadolinium-based contrast agent, we obtained T1W, fast-spoiled gradient echo sequences with fat suppression (CET1WFS) for axial, coronal, and sagittal images. We applied a 260 × 260 mm FOV, 320 × 320 matrix, and 2 mm slice thickness; in addition, a 3 mm slice thickness was reconstructed in all three planes. The injection rate was 2 ml/s (using a power injector), with a dosage of 0.1 mmol/kg body weight gadobenate dimeglumine (Multihance, Bracco, Italy). The MPMRI examination lasted for 30 min.

FDG-PET-CT scans were performed after at least 6 h of fasting and were carried out in compliance with the standard whole-body protocol using a Biograph 6 HD type camera (Siemens, Knoxville, TN). The injected 18F-FDG had radioactivity in the range of 3.7 to 5.5 MBq/kg. The time from FDG injection to PET data acquisition was between 55 and 120 min. In each case, patients were tested for blood sugar level using blood taken from the fingertip before administering the FDG. For patients with glucose level higher than 7 mmol/l (126 mg/dl), the scan was rescheduled. PET/CT scanning took place from the skull base to the upper third of the thighs. Non-enhanced, low-dose (110/70 kV/mAs) CT was performed for all patients. PET data acquisition lasted for 3 min for each bed position. Attenuation-corrected and non-corrected images were reconstructed with the help of the CT data using a 3D iterative algorithm. A manufacturer-specific implementation of the point spread function (PSF) correction method (True-X) was also applied during reconstruction, which helped to recover the activity concentration of smaller lesions as well as provide uniform spatial resolution throughout the FOV. The degree of suspicion of malignant involvement was based on qualitative visual interpretation of the images, and no quantitative analysis, such as the measurement of standardised uptake values, was performed.

The imaging data of the MRI and PET/CT examinations of the patients were compared to the pathological findings. Based on the 2^nd^ edition of the Medical statistics, the sensitivity, specificity, positive predictive value, accuracy and “Likelihood Ratio” (LR) of the examinations were calculated [19].

## Results

Thirty eight patients were examined using both PET/CT and wide-bore 3T MPMRI. Twenty four (63 %) patients were male, and 14 (37 %) were female. The mean age of the study group was 63 years, with a range between 24 and 78 years. All patients had carcinoma proven by cytology after FNAB from a cervical LN metastasis before PET/CT and MRI.

**Fig. 1:**
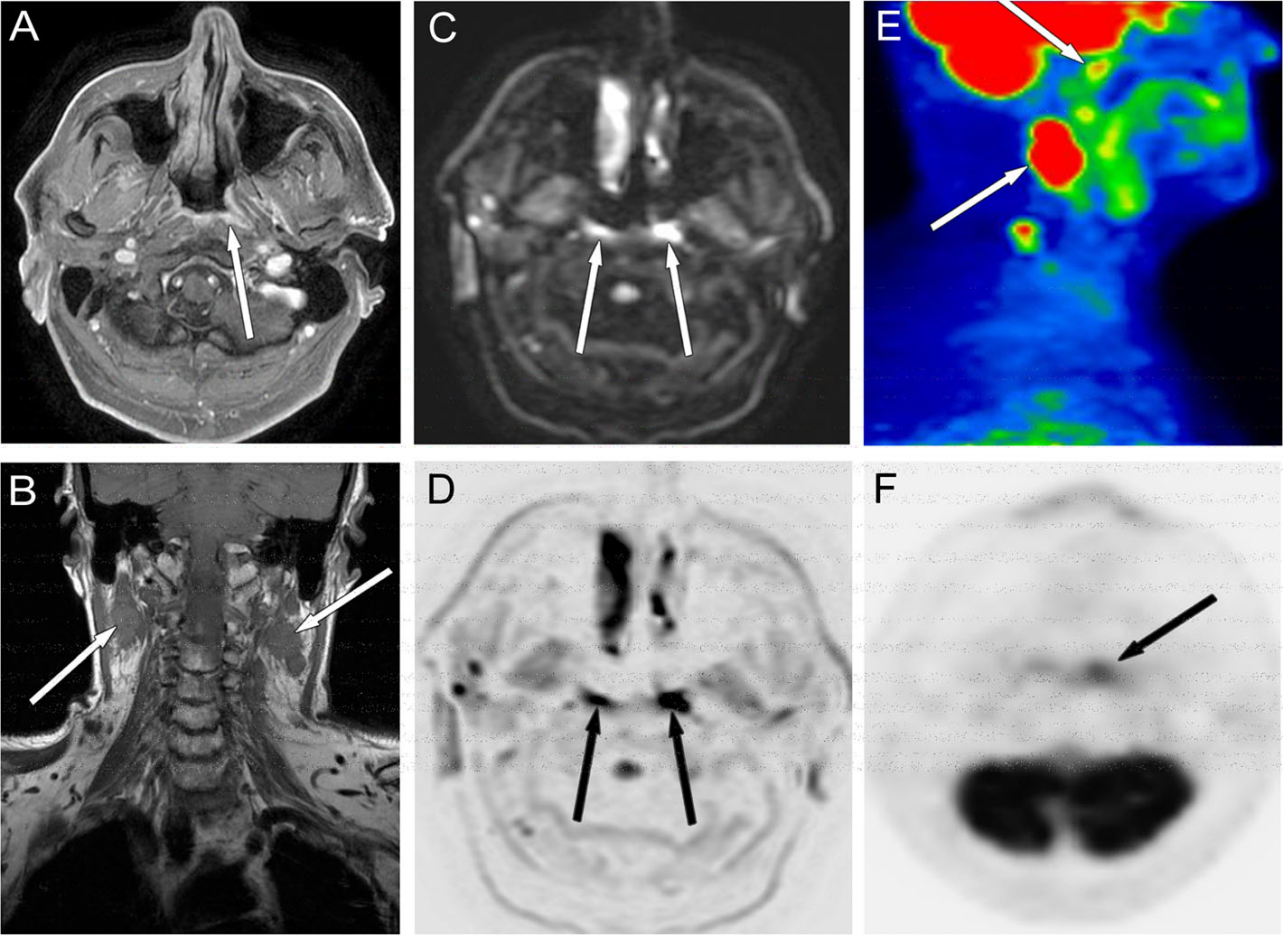
Images collected from a 64-year-old male with T1 nasopharyngeal carcinoma and bilateral metastatic nodes. Axial contrast-enhanced T1-weighted fat suppression (CET1WFS) image (**a**) shows a small enhancing lesion in the left side of the nasopharynx (*arrows*). Coronal T1W image (**b**) shows bilateral LN involvement. Bilateral diffusion restriction with signal increase can be seen in the axial trace DW images (**c** and **d**, *b*-value: 1,000 s/mm^2^, ADC: left 0.772, right 0.758 ×10^−3^mm^2^/s in the PT and 0.991 ×10^−3^mm^2^/s median ADC value was found in the LNs). Increased uptake corresponding to the occult primary tumour (*arrow*) can be seen on the sagittal PET/CT fusion image (**e**) of metastatic nodes (*lower arrow*) as well as on the axial FDG-PET scan (**f**). SCC of nasopharynx, stage T1N2M0

**Fig. 2:**
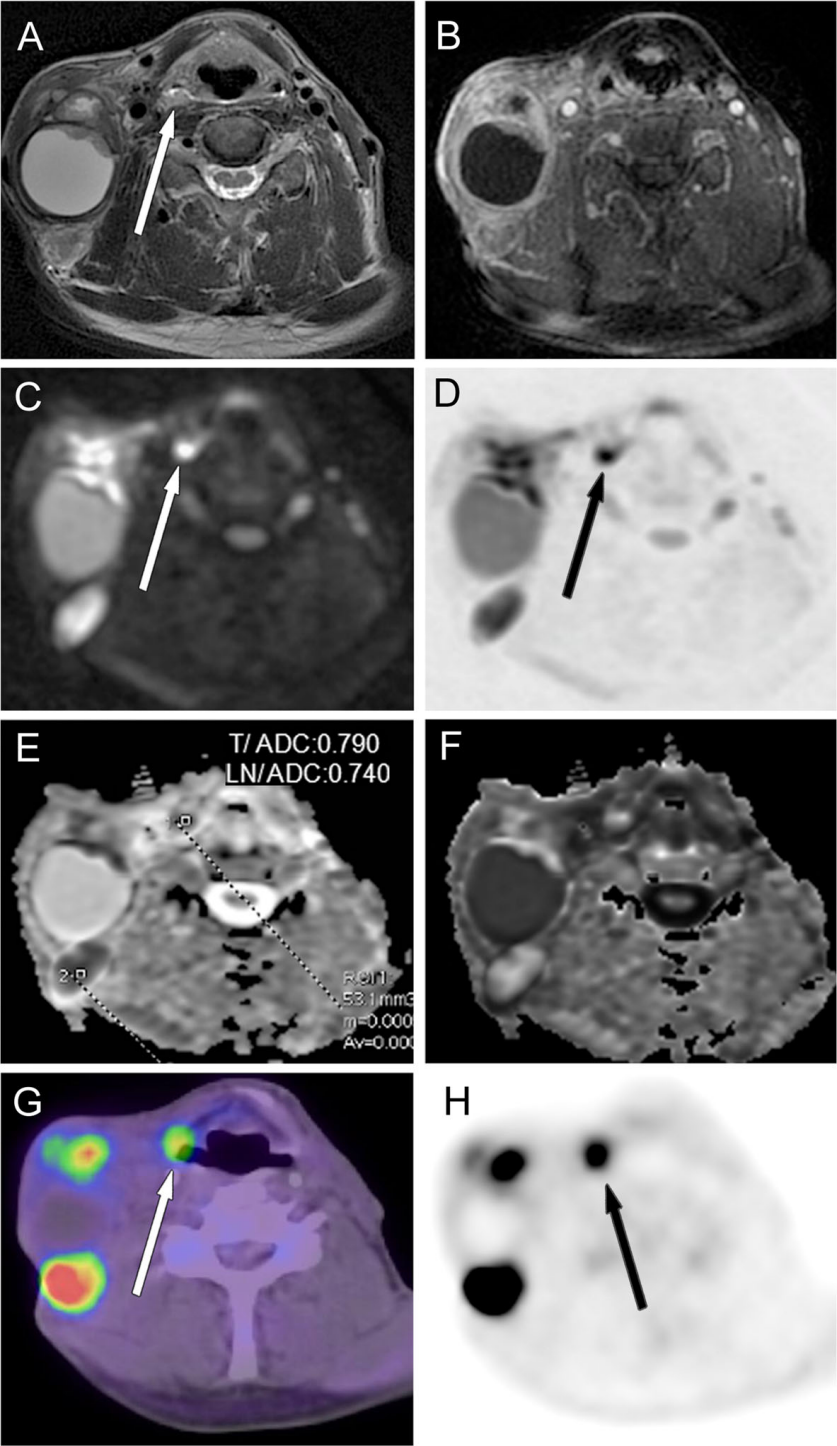
Images of a 56-year-old male with a large right-sided LN metastasis of a small SCC in the right piriform recess. T2W-STIR (**a**) and CET1WFS (**b**) show small soft tissue swelling in the entrance of the right piriform recess; ipsilateral kolliquated LNs can be observed. Findings also revealed substantial diffusion restriction with signal increase in axial trace DW images (**c** and **d**, *b*-value: 1,000 s/mm^2^) and a corresponding signal decrease in the ADC map (**e**) and a signal increase in the e-ADC image (**f**). Note the ADC values, which measured 0.790 × 10^−3^ mm^2^/s in the PT and 0.740 × 10^−3^ mm^2^/s in the LN. On the axial PET-CT fusion image (**g**) and on the FDG-PET scan (**h**), increased uptake corresponding to the occult PT (*arrow*) and metastatic nodes can be seen. SCC of hypopharynx, stage T1N3M0

Possible PT sites were indicated by PET/CT in 24 patients and by MPMRI in 21 cases. Of these patients, 17 PET-CT and 15 MRI examinations confirmed histologically malignant tumours (Table 1 shows the sites of the PTs, Figs. 1 and 2). The detection rate of conventional native MRI was 15.8 %, of conventional native sequences plus DWI 36.8 %, of conventional native sequences with CET1WFS 39.5 %, of MPMRI 39.5 %, and of PET/CT 44.7 %.

**Table 1:** Localisation of detected CUP with LN metastasis in the neck region

Tumour sites	MPMRI			PET-CT			Histology
	T+	F-	F+	T+	F-	F+	
Nasopharynx	3			3		1	3
Mesopharynx	2			2			2
Palatine tonsil	2		1	2		1	2
Base of tongue	3		3	2		5	3
Glandula submand.			1				
Hypopharynx	2			2			2
Supraglottic larynx	1			1		1	1
Parotis	1			1			1
Thyroid			1			1	
Neck soft tissue		1			1		1
Lung	1			1			1
Breast		1		1			1
Colon - sigma				1			1
SUMMARY	15	2	6	17	1	7	18

*T+* true positive, *F-* false negative, *F+* false positive

**Fig. 3:**
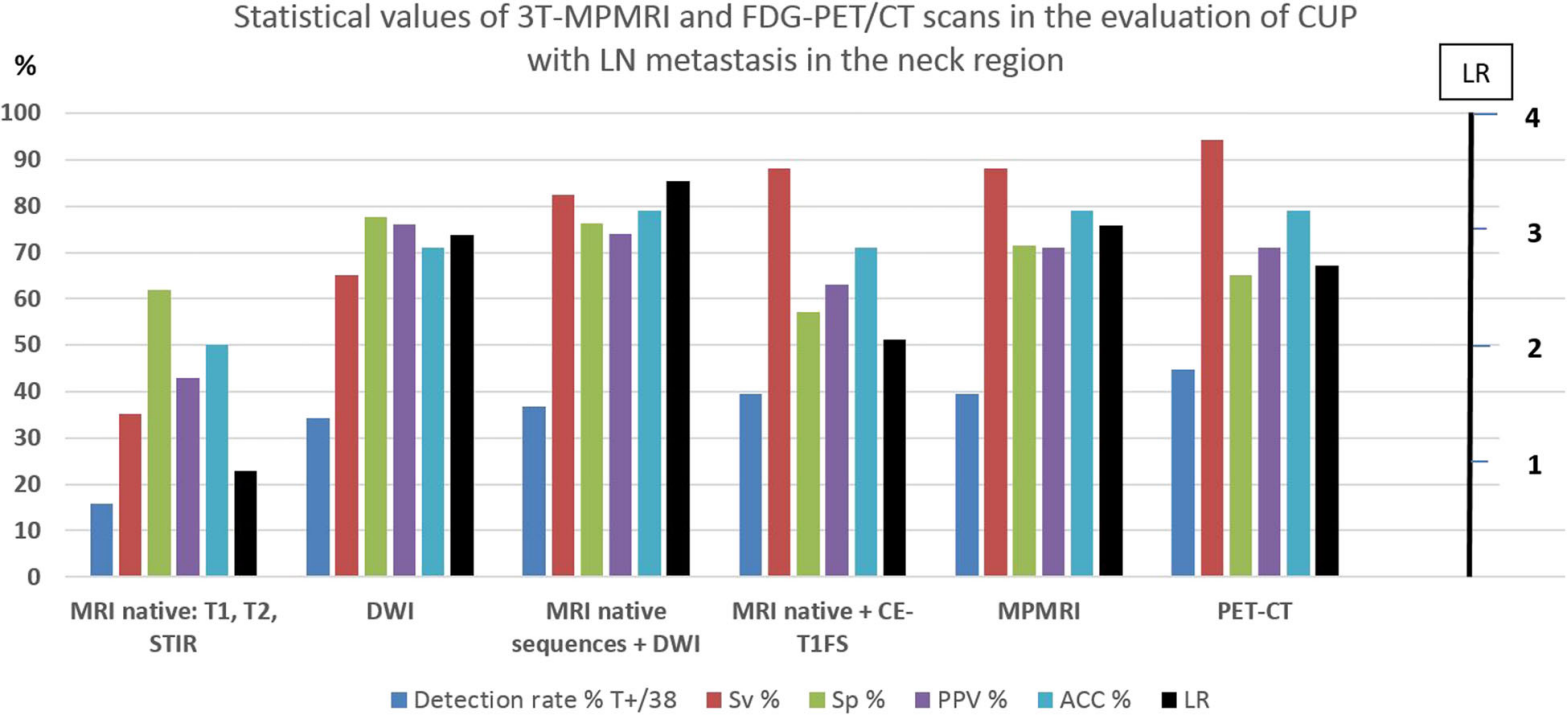
Statistical values of 3T MPMRI and FDG-PET/CT scans in the evaluation of CUP with LN metastasis in the neck region

In the complex diagnostic evaluation procedure (imaging + panendoscopy + biopsy), a total of 18 patients (detection rate 47.4 %) received histologically confirmed PT diagnoses. Table 2 and Fig. 3 show the statistical values.

**Table 2:** Statistical values of 3T-MPMRI and FDG-PET/CT scans in the evaluation of CUP

	Detection rate % T+/38	T+	F-	Sv %	T-	F+	Sp %	PPV	ACC %	LR
MRI native: T1, T2, STIR	15.8	6	11	35.3	13	8	61.9	0.43	50.0	0.92
DWI	34.2	13	2 + 5^a^	65.0	14	4	77.8	0.76	71.1	2.95
MRI native sequences + DWI	36.8	14	3	82.4	16	5	76.2	0.74	79.0	3.42
MRI native + CE-T1FS	39.5	15	2	88.2	12	9	57.1	0.63	71.1	2.05
MPMRI	39.5	15	2	88.2	15	6	71.4	0.71	79.0	3.03
PET-CT	44.7	17	1	94.4	13	7	65.0	0.71	79.0	2.69
Overall proved by histology	47.4	18								

*T+* true positive, *F-* false negative, *T-*: true negative, *F+* false positive, *Sv* sensitivity, *Sp* specificity, *PPV* positive predictive value, *LR* Likelihood Ratio

^a^in 5 cases reduced imaging information because of susceptibility artifacts

The PT stage determined in the neck based on imaging was T3 only in 1 case of a sub-mucous cancer in the mesopharynx. T2 in 5cases and in the other 9 neck cases, the tumour was classified as T1. The lung and the breast cancers were T2 and the colon cancer was T3 stage.

**Fig. 4:**
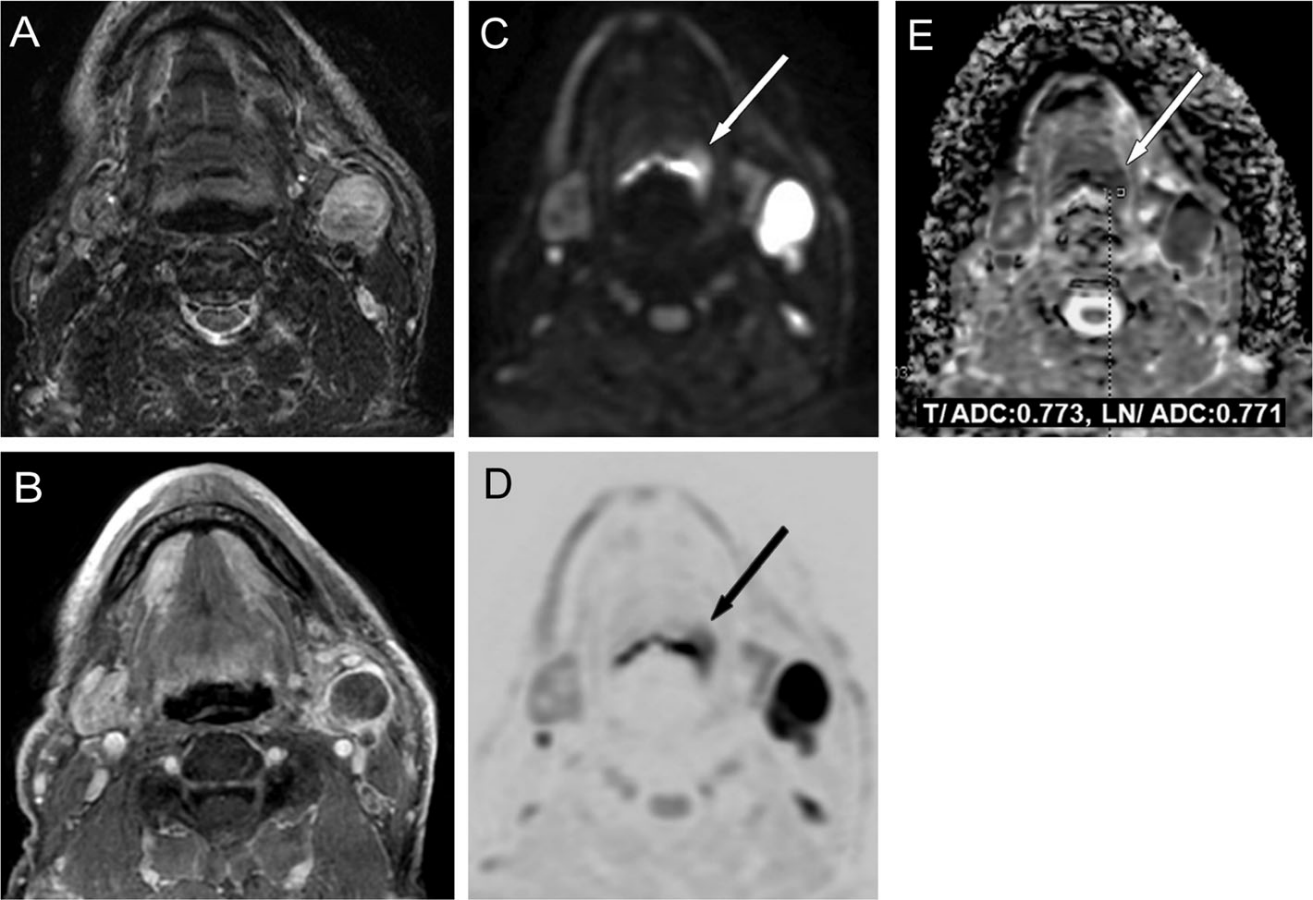
Images obtained from a 77-year-old male illustrate false negative conventional native MRI (**a** T2W-STIR). True positive findings showing a small signal enhancement with contrast (**b** CET1WFS), increase with diffusion restriction can be seen in the axial original DW image (**c**, **d**, *b*-value: 1,000 s/mm^2^) and the corresponding signal decrease in the ADC map (**e**), which measured 0.773 × 10^−3^ mm^2^/s in the PT and 0.771 × 10^−3^ mm^2^/s in the LN. SCC of mesopharynx, stage T1N2bM0

The histologically proven tumours typically demonstrated high signal intensity on high-b-value DWIs with decreased signal intensity on the ADC (Fig. 4), as well as high signal intensity on the e-ADC map (Fig. 2). The median ADC value was found to be 0.892 × 10^−3^mm^2^/s for the neck PTs. Table 3 shows the ADC values, stage and histology of the histologically proven occult PTs with cervical node metastasis in the head and neck.

**Table 3:** ADC values, stage and histology of the histologically proven occult PTs

Sites	ADC 10^−3^ mm^2^/s	Stage	Histology
1. Nasopharynx	0.760	cT2 cN3b	Poorly differentiated SCC
2. Nasopharynx	0.798	cT1 cN2	Nasopharyngeal carcinoma-EBV associated
3. Nasopharynx	0.772	cT2 cN3b	Poorly differentiated SCC
4. Mesopharynx	1.150	cT1 cN2b	Poorly differentiated SCC
5. Mesopharynx	artefacts	cT1 pN2b	Poorly differentiated SCC
6. Base of tongue	0.773	cT1 pN2a	Well differentiated SCC
7. Base of tongue	1.20	cT2 pN2b	Moderately differentiated SCC
8. Base of tongue	0.816	cT2 pN2a	Poorly differentiated SCC - HPV associated
9. Palatine tonsil	0.821	pT1 pN2b	Poorly differentiated SCC
10. Palatine tonsil	0.702	cT3 cN2b	Moderately differentiated carcinoma planocellulare
11. Hypopharynx	1.170	cT1 pN3	Poorly differentiated SCC
12. Hypopharynx	0.790	cT1 cN3	Poorly differentiated SCC
13. Supraglottic larynx	0.882	cT1 cN3	Poorly differentiated SCC
14. Parotis	0.908	cT1 cN3	Moderately differentiated carcinoma planocellulare
15. Neck soft tissue^b^	0.943	pT2 pN0	Hemangiosarcoma
16. Base of tongue^a^	2.1	T0NX	NO sign of malignancy

*SCC* squamous cell carcinoma

^a^Fine needle aspiration cytology of a neck nodal mass was false positive for cancer

^b^Neck mass was considered by cytology and by both imaging methods metastatic nodal conglomerate

**Fig. 5:**
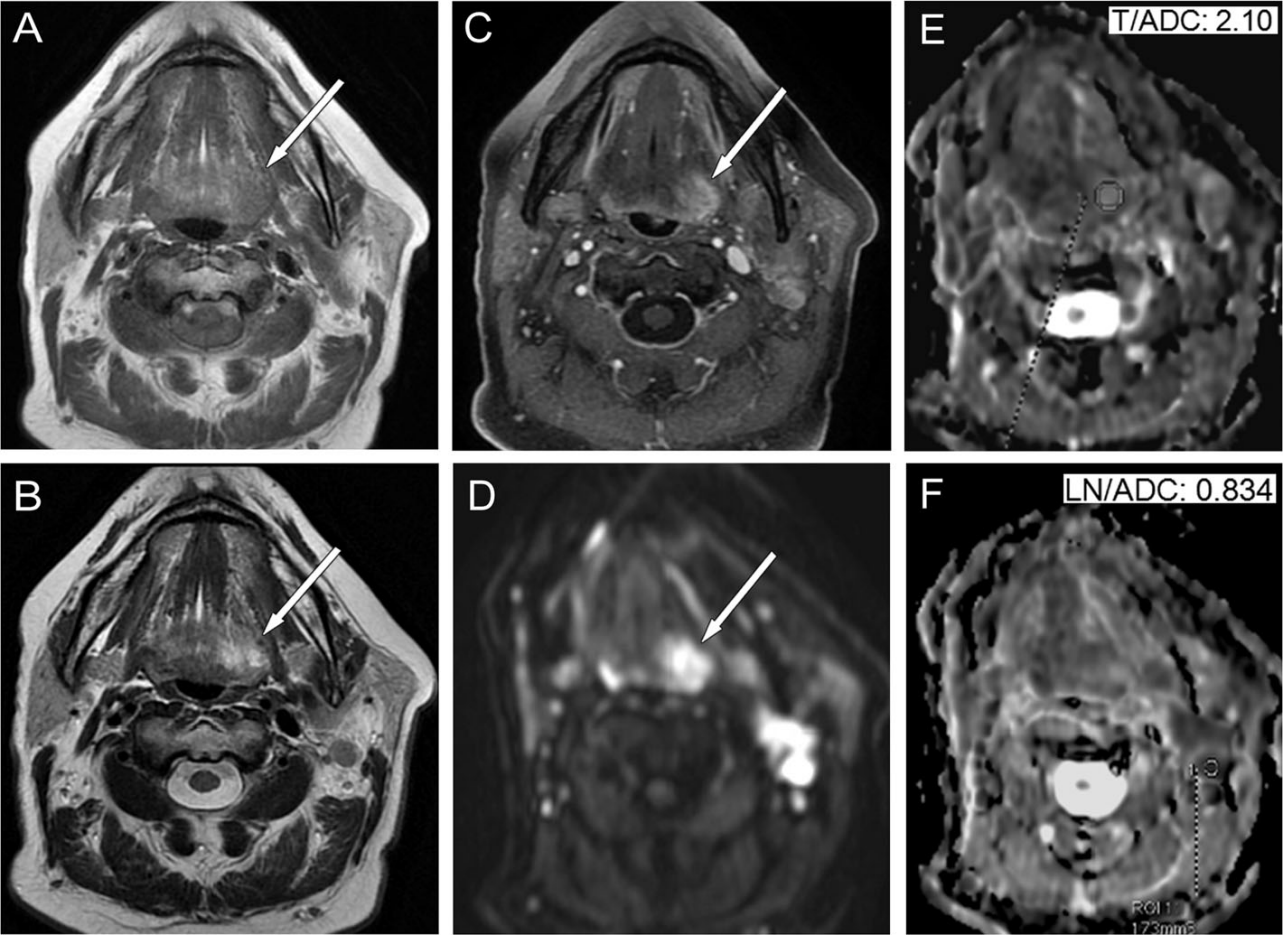
Images obtained from a 54-year-old female illustrate a false positive interpretation error (*arrows*) on conventional MRI scans (**a** T1W, **b** T2W, **c** CET1WFS). On the axial trace DW image (**d**, b-value: 1,000 s/mm^2^), a signal increase can be seen without diffusion restriction on the ADC map (**e**), which measured 2.100 × 10^−3^ mm^2^/s in the radix linguae corresponding to inflammation and 0.834 × 10^−3^ mm^2^/s in the LN (**f**). CUP, SCC, stage TXN2bM0

In a histologically proven case of inflammatory disease, all MR measurements were false positives for tumour of the base of tongue, except for the DWI, with an ADC value of 2.100 × 10^−3^mm^2^/s (Fig. 5). In 3 base of tongue cases all MR sequences were false positive, DWI demonstrated by the high signal intensity on high-b-value, decreased signal intensity on the ADC map, and resulted 0.928, 0.741, 1.13 × 10^−3^mm^2^/s ADC values.

In 5 cases the PT sites could not be evaluated on DWI secondary to the reduced information because of susceptibility artefacts.

Nodal (N) staging was also carried out, and both methods were positive for cervical LN metastases in every patient. The upper and middle cervical LNs were the most frequently involved nodal levels.

Two patients, metachronous metastases were found with PET/CT, one poorly differentiated SCC manifestations in the lung and in the bone, and lung metastases were detected in the hemangiosarcoma case.

Thirty two patients received radiotherapy, 26 combined with chemotherapy (Table 4).

**Table 4:** Therapies administered for clinically occult 36 PTs with neck node metastasis

Tumor sites	RT	RT+ ND	CT	CT + RND	CRT	CRT + ND	MND	Sum	+ Primary Tumor resection
Nasopharynx					3			3	
Mesopharynx	1					2		3	1 tumor resection
Base of tongue						2^b^		2	
Palatine tonsil					1	1		2	1 tonsillectomy
Hypopharynx			1 +			1		2	
Larynx					1			1	
Parotis					1			1	parotidectomy
Neck soft tissue						1		1	tumor resection
Lung					1			1	lobectomy
Breast			1					1	
Colon / sigma					1			1	
Final CUP	2	3^a^		1	4	7^b^	1++	18	
	3	3	2	1	12	14	1	36	

In one patient the lymph node cytology was false positive proven by the further examinations. One patient refused the treatment

*CUP* cancer of unknown primary, *ND* neck dissection, *RND* radical ND, *MND* modified ND, *SND* selective ND, *RT* radiotherapy, *CT* chemotherapy, *CRT* chemoradiotherapy

^a^1 RND, 1 SND, 1 MND **+** patient refused RT

^b^1 MND other RND **++** wait and see follow up

For radiation therapies planning both modalities were effectively applicable. Patients were immobilized with a thermoplastic mask and treated with 3-dimensional (3D) conformal treatment or intensity-modulated radiation therapy (IMRT) using 6 MV photons. The planned radiation dose to the primary tumor and the involved lymph nodes was 66–70 Gy in definitive radiotherapy. For chemoradiotherapy, 100 mg/m^2^ cisplatin was administered two or three times (on the 1^st^, 22^nd^and 43^rd^days of radiotherapy).

One patient with N1 CUP after selective neck dissection (SND) was followed by clinical examination and imaging and did not receive any other therapy. Nineteen patients underwent neck dissection (ND), 14 radical ND (RND), 1selective ND (SND) and 4 modified ND (MND).

## Discussion

Detection of the PT has a large impact on the prognosis of CUP patients with metastatic LNs in the neck. As in most cancers, the prognosis mainly depends upon the stage of the tumour and the applied therapy [20]. Superior characterisation of soft tissues and better visualisation of tumour extent can be of benefit both in surgical and radiotherapy planning by improving the assessment of planning margins for dose escalation [21, 22].

The location of metastatic LNs may indicate the location of the unknown PT. When LNs of the upper and middle cervical level are involved, the primary site of the tumour is more likely located in the HN region. If lower cervical levels are involved, the PT is often located below the clavicles. In patients in whom histopathology has shown SCC, the primary site is often located in the palatine tonsils or in areas difficult to inspect, such as the piriform sinus or the nasopharynx [23, 24]. Lapeyre et al. reported on 87 patients with nodal neck metastasis and an unknown primary site who were evaluated between 1969 and 1992 and underwent a unilateral tonsillectomy; 26 % of these patients were found to have tonsillar cancer [25]. In our patients the number of cases of histologically proven palatine tonsil carcinoma was only 2 (5.3 %).

The sites of distant metastasis (M) in most HN cancer (HNC) cases are the lung and the bone (and rarely the liver and brain). The risk of distant metastasis is more dependent on the “N” than on the “T” status [8, 26]. In our study we found 3 patients with synchronous distant metastases using PET/CT. In 1 unknown primary tumour with N3 stage patient occult bone metastasis was detected. In case of colon PT synchronous bone-, lung- and liver metastases were found. In the triple negative breast cancer patient multiple manifestations were found in the thorax wall and in the abdomen.

In the management of CUP patients, clinical examination with panendoscopy is the basic method, although the majority of suspected primary sites are detected by imaging workup. MRI is considered superior to CT for obtaining a better assessment of the PT in the HN region. Panendoscopy with imaging-directed biopsy to suspected PT sites or to the most frequent sites of occult tumour can detect CUP. If the metastatic nodes are in the upper and/or middle cervical levels and non-invasive diagnostic tools are negative for CUP, tonsillectomy might be the diagnostic and therapeutic choice, especially in the case of SCC [27]. In our study, none cancer-positive tonsillectomy cases were found after non-invasive diagnostic tools failed to detect the CUP.

In the literature, several publications describe the efficacy of FDG-PET/CT in detecting CUP with cervical LN metastases [10, 28, 29]. The increased glycolysis of malignant cells can be detected with PET using 18F-labeled FDG, and FDG-PET was shown to change the clinical management of one-third of CUP patients [30].

In our study, we found similar detection rates of primary sites when using 3T MPMRI (39.5 %) and PET-CT (44.7 %). PET/CT showed higher sensitivity (Sv: 94.4 %) but lower specificity (Sp: 65.0 %) in detecting the PT site compared to 3T MPMRI (Sv: 88.2 %, Sp: 71.4 %). DWMRI did not improve the sensitivity, but increased the specificity of MPMRI.

Srinivasan et al. verified that DWI can also be successfully used on 3T machines. These authors reported a mean ADC value of 1.101×10^−3^ mm^2^/s in the characterisation of HNSCC [31, 32]. Studies have also evaluated the influence of magnetic field strength on ADC values and reported no statistically significant difference in HN tumours between the ADC values at 1.5 and 3T [33, 34]. Moreover, ADC values are independent of magnetic field strength [35].

In our patient group, the mean ADC value of histologically proven PTs was 0.892×10^−3^mm^2^/s. Inflammatory tissue with a high fluid content can result in a signal increase, not only in lower b-value images but also in high b-value images (similar to tumorous tissue), because of incomplete suppression of the signal (the so-called T2 shine through phenomenon). However, the increased ADC value allows for differentiation between inflammation and tumour; in a case of suspected tumour on conventional MR sequences, quantitative evaluation of ADC (2.1×10^−3^mm^2^/s) could be used to determine the accurate diagnosis of inflammation (Fig. 4).

SCC in the palatine tonsil can be challenging it is rarely necrotic and typically shows a homogeneous appearance similar to normal lymphoid tissue in the tonsil when viewed using conventional MRI [36]. In our study, the lingual tonsil was the most challenging site, often pitfalls in our CUP evaluation. We evaluated 3 false positives beside 3 true positives with MPMRI and 5 false positives and 2 true positives with PET/CT in the base of tongue region. Diffusion can also be restricted in non-tumorous lingual tonsil tissue, and DWI by itself is thus not sufficient for tumour differentiation in normal sized and -shaped lingual tonsil. In the 3 false positive base of tongue cases DWI demonstrated by the high signal intensity on high-b-value, decreased signal intensity on the ADC map, and resulted 0.928, 0.741, 1.13×10^−3^mm^2^/s ADC values.

Detection of the primary cancer site before the start of treatment is of great significance because this location reduces unwanted side effects [37, 38]. In a previous study, the 3-year survival rate of patients with detected HN PTs was 100 % after selected treatment, as compared to 58 % in patients with elected therapy for unknown PT tumours [39].

Our cancer patients 32 received radiotherapy (66–70 Gy), partly combined with chemotherapy (26 patients). In cases of detected primary, 3D-conformal IMRT was applied due to its better technical capacity, to reduce the dose delivered to normal tissues because of its improved focus on the precision of target volumes.

The patients’ follow-up care included a follow-up visit with the HN specialist every three months, an MRI examination every six months, and an annual PET/CT examination. In the past three and half years, only 3 patients (out of 38) have died, 2 because of extended tumour spread, one in the triple negative breast carcinoma, and one in the lung adenocarcinoma, the third patient withT3N2b stage palatine tonsil cancer because of vascular, myocardial attack. There was no case in which the PT appeared after radical therapy. About one year after CRT in 3 patients appeared recurrent neck node metastases, in all the three cases the initial N stage was N3.

### Limitations

Our study, however, had some limitations. First, this was a retrospective study covering only a 3.5-year period. Second, the number of patients was small (*n* = 38), and therefore the results and correlations should be interpreted with caution.

## Conclusions

In cases with highly probable PT sites detected by either MPMRI or FDG-PET/CT, imaging may provide clinicians with valuable information on where to obtain a tissue sample.

Because conventional native sequences with DWI, 3T-MPMRI and PET/CT were similarly accurate (Acc: 79.0 %) and had similar likelihood ratio (LR: 3.42, 3.03, 2.62) for detecting unknown PT sites, both imaging methods may be used to determine the final diagnosis of patients with CUP in the HN region. DWMRI by its increased specificity (Sp: 77.8 %) and likelihood ratio (LR: 2.95) was shown to be capable of differentiating tumour from non-tumorous tissue. Disadvantage of DWI is its high sensitivity to the motion artefacts (5/38, 13.2 %).

The sensitivity of FDG-PET/CT (94.4 %) is higher than MPMRI (88.2 %), and this statistical indicator in case of finding CUP is more important, than the specificity of the modalities.

Although MPMRI has the advantage of being radiation free, but this is hardly a concern in this patient group as most of the patients get radiation therapy.

While using PET/CT whole body information can be obtained in one examination along with information about metastases, and second malignancies.

MPMRI shows the local soft tissue status and the connection of the primary tumor with the surrounding organs more accurately.

Summarizing the above, mentioned results, in case of CUP PET/CT should be the first method of choice if it is available.

After clinically proved positive PET/CT the additional MPMRI can clarify the exact primary tumor stage, it can be advantageous in clarifying the prognostic factors, which is necessary in case of advanced tumor stage and when surgery is under consideration.

In case low N stage is likely after the clinical examination and *wait and see policy* can be considered, MPMRI is recommended, and in this case the significance the of radiation free MPMRI is increasing.

In cases with PT-positive imaging results during CUP evaluation, the next step should be panendoscopy with biopsies.

For radiation therapies planning both imaging modalities are effectively applicable.

The head and neck imaging is a challenging area which needs strict protocols, special attention and well trained specialists, radiologists and radiographers as well.

## Additional file

Additional file 1: Table 5. Data collection. (XLSX 22 kb)

## Abbreviations

18F-FDG: 18F-fluorodeoxyglucose
3T: 3-Tesla
Acc: Accuracy
ADC: Apparent diffusion coefficient
CEMRI: Contrast-enhanced MRI
CET1WFS: Contrast-enhanced T1-weighted with fat suppression
CT: Computed tomography
CUP: Cancer of unknown primary origin
DWI: Diffusion-weighted imaging
DW-MRI: Diffusion-weighted MRI
FDG: Fluoro-2-deoxy-D-glucose
FNAB: Fine needle aspiration biopsy
HN: Head and neck
HNC: Head and neck cancer
HNSCC: Head and neck squamous cell carcinoma
LN: Lymph node
LR: Likelihood ratio
MND: Modified neck dissection
MPMRI: Multiparametric – MRI
MRI: Magnetic resonance imaging
ND: Neck dissection
PET/CT: Positron emission tomography - computed tomography
PT: Primary tumour
RND: Radical neck dissection
ROI: Region of interest
SCC: Squamous cell carcinoma
SND: Selective neck dissection
Sp: Specificity
Sv: Sensitivity
